# Emergency physicians in pain medicine: Workforce trends, competency overlap, gaps, and opportunities for integration

**DOI:** 10.1016/j.inpm.2025.100722

**Published:** 2025-12-17

**Authors:** Jeffrey R. Merz-Herrala, Felipe Ocampo, Christopher R. Abrecht, J. Ben Arevalo, Nu Cindy Chai

**Affiliations:** a Department of Anesthesia & Perioperative Care, Division of Pain Medicine, University of California, San Francisco, CA, USA; b Department of Emergency Medicine, University of California, San Francisco, CA, USA

**Keywords:** Fellowship, Multidisciplinary, Competency, ACGME, ABEM

## Abstract

**Background:**

Pain is a leading complaint in Emergency Department (ED) visits, yet historically, few Emergency Physicians (EPs) have pursued fellowship training in Pain Medicine. In recent years, however, applications from EPs have risen sharply, contrasting with declines in other specialties. Despite this growth, there has been no systematic analysis of how Emergency Medicine (EM) training overlaps with the required competencies of the Pain Medicine fellowship. To our knowledge, this study represents the first such effort.

**Methods:**

We systematically compared the Accreditation Council for Graduate Medical Education (ACGME) Program Requirements for Pain Medicine with five core EM training documents: the ACGME Program Requirements for EM, ACGME EM Milestones, ACGME Key Index Procedures, ACGME Procedure Logs, and the American Board of Emergency Medicine (ABEM) EM Model of Clinical Practice. Each ACGME Pain Medicine Program Requirement was evaluated by a group of Pain and EM physicians for its degree of overlap with these EM training frameworks and categorized as having significant, partial, or minimal overlap in competency.

**Results:**

EM training exhibits a strong overlap with Pain Medicine in patient care, encompassing neurologic and musculoskeletal evaluation, psychiatric assessment, and the diagnosis of acute and chronic pain. EPs also demonstrate procedural strengths in airway management, intravenous access, ultrasound-guided interventions, life support, procedural sedation, managing emergencies, along with medical knowledge in acute pain management, medication detoxification, and treatment of substance use disorders. Gaps were identified in the interpretation of electrodiagnostic studies, advanced imaging, prescription of rehabilitation strategies, long-term opioid management, and advanced fluoroscopic and neuromodulation procedures. These findings highlight EM's strong foundation in acute care and procedures, while clarifying domains that require targeted fellowship training.

**Conclusions:**

EPs contribute valuable skills to Pain Medicine but require structured opportunities to address predictable training gaps. Electives, mentorship, and flexible curricula may help bridge these deficiencies.

## 1 Introduction

Pain is the leading complaint in over half of ED visits, with chronic pain accounting for 16 % [[1], [2], [3], [4], [5]]. EPs manage pain across a wide range of conditions—from cancer and sickle cell disease to spine pathology, migraines, and trauma. Despite this overlap, relatively few EPs have historically pursued fellowship training in Pain Medicine. As of early 2025, only 55 physicians held Pain Medicine subspecialty certification, accounting for 1.2 % of the 4773 subspecialty credentials granted by ABEM—the fewest of any recognized field [6].

Pain Medicine has long been multidisciplinary, commonly drawing fellows from Anesthesiology, Physical Medicine and Rehabilitation (PM&R), Neurology, and Psychiatry. The ACGME formally added EM to this group in 2014, recognizing the relevance of pain training for EPs [7]. Since that time, the applicant pool has shifted considerably: between 2019 and 2023, Pain fellowships saw a 45 % decline in Anesthesiology applicants and a 15 % decline overall, while applications from EM increased nearly 300 %—the most significant growth of any base specialty [7,8].

This rapid growth is likely due to several factors. EM physicians may seek continuity and diagnostic depth that are not always available in the ED, while others may be drawn to procedural overlap, including ultrasound-guided injections, regional anesthesia, and suturing [[9], [10], [11], [12]]. Workforce pressures, including a projected oversupply of EPs by 2030, further incentivize subspecialization [13]. Burnout—affecting nearly half of EPs due to high acuity and irregular hours—also likely plays a role, with Pain Medicine's outpatient structure offering schedule stability and longitudinal care [10,11,[14], [15], [16], [17]]. Together, these factors may explain the rise in EM applications to Pain Medicine and raise key questions: which competencies are addressed in EM training, where gaps remain, and how programs can best support integration.

Prior work by Snyder and Solis introduced the conceptual overlap between EM training and Pain Medicine [18,19]. Building on this, we provide the first systematic analysis comparing ACGME Pain Medicine requirements with core EM training frameworks. Our findings identify areas of overlap and underexposure in patient care, medical knowledge, diagnostic interpretation, and procedural skills. We conclude with recommendations for training programs.

## 2 Material and methods

We conducted a qualitative synthesis of competency documents and relevant literature to identify areas of overlap between EM training and ACGME Pain Medicine requirements.

### 2.1 Review group

The reviewers comprised a multidisciplinary group of physicians representing EM, anesthesiology, neurology, pain medicine, and palliative care. All had direct experience in an ACGME-accredited Pain Medicine Fellowship program, an EM residency program, or both.

### 2.2 Competency documents

The ACGME Program Requirements are a set of standards designed to ensure the quality of graduate medical education across residency and training programs. Individual specialties (Pain Medicine and EM) describe specific competencies in areas including patient care, procedural skills, and medical knowledge [20,21]. In this analysis, the 2025 ACGME Program Requirements for Pain Medicine were systematically compared with five primary EM training framework documents: (1) the ACGME EM procedural requirements, (2) ACGME EM procedure log data, (3) the ACGME Program Requirements in Emergency Medicine, (4) the ACGME Emergency Medicine Milestones, and (5) the Model of the Clinical Practice of Emergency Medicine from the American Board of Emergency Physicians (ABEM) [[21], [22], [23], [24], [25]]. Sections 4.3–4.9 of the ACGME Pain Medicine Program Requirements (Professionalism, Practice-Based Learning and Improvement, Interpersonal and Communication Skills, Systems-Based Practice) were excluded, as these domains are uniform across specialties and not specific to EM and Pain Medicine.

### 2.3 Process

Each Pain Medicine competency was listed in full and examined for overlap with EM frameworks. Overlap was categorized as *green* (significant competency overlap), *yellow* (partial competency overlap), or *red* (no significant overlap). An initial review was conducted independently by two reviewers; any disagreements were adjudicated by a senior physician in Pain Medicine. A supplementary table provides the complete set of Pain Medicine competencies with corresponding alphanumeric references to EM requirements, Milestones, and ABEM Model domains, serving as the evidentiary basis for all ratings (Supplementary Table 1).

### 2.4 Literature synthesis

To contextualize these findings, we also conducted a narrative review of the literature on broader workforce trends, EM trainee motivations, and strengths/weaknesses not captured by the training competency documents. This synthesis informed our analysis of competency overlap and our discussion of opportunities for integration between EM and Pain Medicine.

## 3 Results

**Fig. 1:**
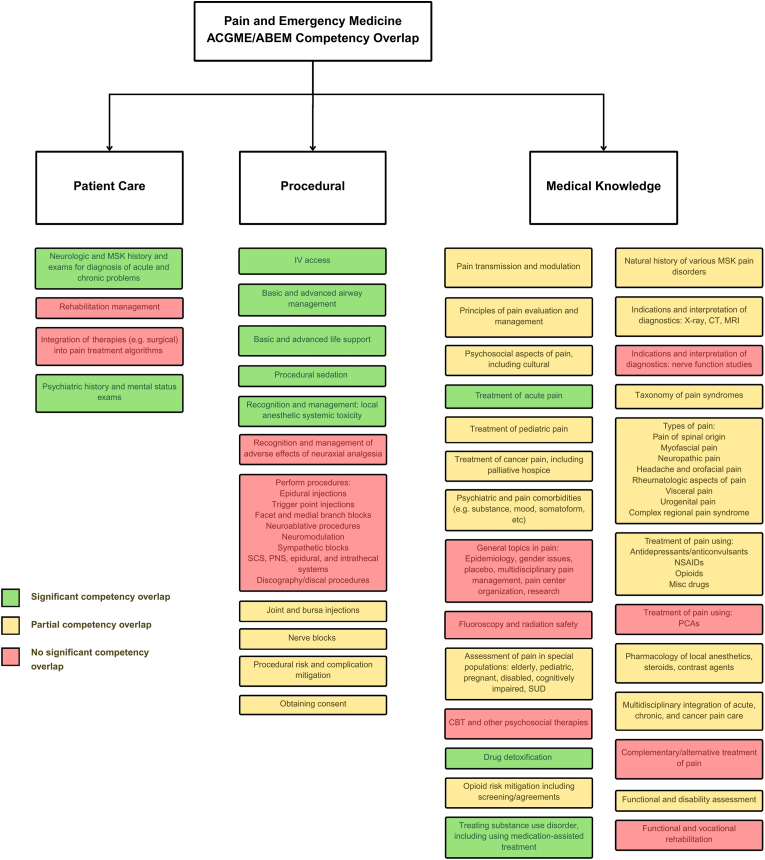
Pain Medicine and EM ACGME and ABEM Competencies Overlap. **Notes:***Sources include ACGME Program Requirements for Graduate Medical Education in Pain Medicine* [20]*; ACGME Program Requirements for Graduate Medical Education in Emergency Medicine* [26]*; ACGME EM Milestones* [22]*; ABEM Model of the Clinical Practice of Emergency Medicine* [23]*. Full review for all ACGME Pain Medicine Competencies provided in*Supplementary Table 1*.* **Abbreviations:** ACGME, Accreditation Council for Graduate Medical Education; ABEM, American Board of Emergency Medicine; US, ultrasound; MSK, musculoskeletal; IV, intravenous; SCS, spinal cord stimulator; PNS, peripheral nerve stimulator; SUD, substance use disorder; CBT, cognitive behavioral therapy; CT, computed tomography; MRI, magnetic resonance imaging; NSAIDs, nonsteroidal anti-inflammatory drugs; PCA, patient-controlled analgesia.

The comparison of EM training frameworks with ACGME Pain Medicine fellowship requirements identified areas of significant, partial, and minimal overlap. Findings are presented across three domains—patient care, procedural skills, and medical knowledge—with a visual summary provided in Fig. 1 and full competency-level detail available in Supplementary Table 1.

### 3.1 Patient care

*Significant overlap (green)*: EM training promotes competency in neurologic, musculoskeletal, and psychiatric examinations, including mental status, cranial nerve, motor, sensory, reflex, cerebellar, and gait assessments. EM physicians are also trained to recognize substance use disorders, address stigma, initiate medication-assisted treatment, and manage acute pain.

*Partial overlap (yellow)*: Training includes experience in eliciting neurologic and musculoskeletal histories, as well as interpreting common CT and MRI findings; however, the fellowship expands this to comprehensive neuromuscular evaluations, advanced brain and spine imaging, and psychosocial integration. Partial overlap also includes identifying candidates for multidisciplinary care, performing complete psychiatric histories with comorbidity assessment, and initiating referrals for psychosocial therapy.

*Minimal overlap (red)*: Competencies not addressed in EM include prescribing spine and musculoskeletal rehabilitation programs, functional and vocational rehabilitation, psychosocial therapies such as cognitive behavioral therapy, complementary and alternative treatments for pain, gender and cultural aspects of pain, epidemiology of pain, and ethics in pain care and research.

### 3.2 Procedural skills

*Significant overlap (green)*: EM training promotes competence in intravenous access, bag-mask ventilation, advanced airway management (laryngeal mask airway and endotracheal intubation), basic and advanced life support, procedural sedation, and recognition and treatment of medical emergencies.

*Partial overlap (yellow)*: Residents perform joint and bursa injections and peripheral nerve blocks, though exposure is variable and limited compared to fellowship training. EM also provides experience in recognizing procedural risks, managing complications, and obtaining informed consent.

*Minimal overlap (red)*: Competencies without EM coverage include fluoroscopically guided epidural injections, trigger point injections, facet and medial branch blocks, neuroablative procedures, sympathetic blocks, discography, spinal cord and peripheral nerve stimulation, intrathecal drug delivery systems, and advanced epidural/intrathecal medication management.

### 3.3 Medical knowledge

*Significant overlap (green)*: EM training provides a strong foundation in acute pain pharmacology, including mechanisms and uses of opioid agonists, antagonists, and mixed agents, as well as prescription drug detoxification.

*Partial overlap (yellow)*: EM curricula cover pain physiology and transmission, the natural history of musculoskeletal disorders, general principles of pain evaluation, and selected pain syndromes (neuropathic, headache, myofascial, visceral, cancer). Foundational knowledge of multimodal analgesics—including NSAIDs, anticonvulsants, and antidepressants—is also present, though the fellowship provides more depth.

*Minimal overlap (red)*: Fellowship medical knowledge competencies not covered in EM include advanced opioid pharmacokinetics, peri-procedural opioid hyperalgesia, neuromodulation mechanisms, integration of surgical approaches for pain, psychosocial risk assessment for permanent procedures, multidisciplinary cancer pain care, organization and management of pain centers, utilization review, pain clinical trial design and interpretation, pain placebo response, animal models of pain, and ethics of experimentation.

### 3.4 Procedure experience from EM residency relevant to pain medicine

Additionally, the ACGME lists procedures required for completion of EM residency, many of which are relevant to Pain Medicine. Table 1 lists the ACGME-required and average number of procedures logged for EM residents in pain-relevant procedures from a 2025 ACGME report [24,25,27].

**Table 1:** EM procedures relevant to pain medicine: Required minimums and average logged counts during residency.

Procedure	ACGME Requirement	Avg. Logged	Relevance to Pain Medicine
Bedside US	150	386	MSK US-guided injections, US-guided intravenous access for challenging access in the clinic
Regional Anesthesia Blocks	10*	9.3*	Peripheral nerve blocks, fascial plane blocks
Lumbar Puncture	15	23	Neuraxial procedures, including intrathecal trials, epidural steroid injections
Procedural Sedation	20	28	Sedation for interventional procedures
Central Lines	20	48	Stellate ganglion and brachial plexus blocks, Seldinger technique
Arthrocentesis	10	Not collected	Diagnostic/therapeutic joint injections
Medical Resuscitation	60	184	Management of medical/procedural emergencies
Laceration Repair	Not a Key Index Procedure	70	Suturing for neuromodulation procedures (intrathecal pumps, spinal cord stimulators)

**Notes:** Sources include ACGME Emergency Medicine Key Index Procedures and Revisions [28]; Trends in Emergency Medicine Resident Procedural Reporting Over a 10-year Period [25]; Procedure Rates Performed by Emergency Medicine Residents: a retrospective review [29]. *Assumes a 4-year residency based on annual averages from Bucher article [29]. Variable depending on the residency program.

**Abbreviations:** ACGME, Accreditation Council for Graduate Medical Education; Avg., average; US, ultrasound; MSK, musculoskeletal.

## 4 Discussion

For EM physicians, our ACGME competency crosswalk demonstrates strong overlap in acute care and specific procedural domains. EPs enter fellowship experienced in advanced airway management, intravenous access, resuscitation, procedural sedation, and recognition of medication, medical, and procedural complications. They are also well trained in detoxification, and treatment of substance use disorders, alongside neurologic and musculoskeletal evaluation and rapid synthesis of complex patient histories. EPs also bring procedural experience in several specific pain-relevant domains: ultrasound-guided needle procedures (via experience in vascular access, arthrocentesis, soft-tissue aspiration and drainage). Many also have basic exposure to regional anesthesia, though the depth of this experience varies substantially by training environment. These strengths provide a meaningful background in specific acute-care and ultrasound-guided skill sets; however, overlap with the core ACGME Pain Medicine procedural requirements remain limited. As our data show, only two of the eight essential techniques demonstrate partial overlap, with the remaining six falling into minimal-overlap domains such as fluoroscopic interventions, neuroablative procedures, and neuromodulation. Thus, the intent is not to suggest that EPs require less procedural training overall, but rather that their existing competencies may allow fellowship programs to devote proportionally less introductory time to these selected acute-care or ultrasound-guided skills and more focused training toward pain-specific procedural domains where overlap is sparse.

Areas where EPs may require reinforcement are concentrated in longitudinal and specialized care. Compared with colleagues from other base specialties, they may have limited experience with continuity of care, long-term opioid management, titration of adjuvant medications, and interpretation of MRI and nerve function studies. Their training also provides minimal exposure to fluoroscopically guided procedures, loss-of-resistance technique, and neuromodulation—core fellowship-level skills in which gaps may be common among entering trainees, regardless of background specialty. Additionally, EPs have limited exposure to rehabilitation prescription, functional restoration, and cognitive-behavioral strategies. While proficient in acute pharmacology, EPs often need additional background in the basic science of pain transmission and modulation. Early, structured exposure to these domains can help ensure EM fellows reach competency parity within the one-year fellowship model.

Our working group also recognized that the ACGME Pain Medicine Program Requirements do not fully capture all skills necessary for practice. EM training confers unique strengths not explicitly reflected in the crosswalk. EPs are accustomed to leading high-stakes conversations—delivering bad news, managing uncertainty, and setting expectations under time constraints—skills that translate to efficient clinic operations, empathic communication, and boundary-setting in complex cases. They are highly skilled at prioritization in multifactorial presentations, which supports agenda setting and shared decision-making when patients present with multiple pain concerns. Efficiency is another defining strength: managing 2–3 undifferentiated patients per hour in the ED prepares trainees to adapt to high-volume clinics [30,31]. Their training also emphasizes interdisciplinary collaboration across a wide range of specialties and allied staff, which mirrors the multidisciplinary nature of pain management. Finally, their experience with fragmented transitions, complications, and psychosocial challenges fosters a systems-level awareness of where care plans may break down or patients may be lost to follow-up, an insight relevant to Pain Medicine practice [32].

### 4.1 Future directions

Building on these findings, future efforts should focus on structured opportunities for exposure, dedicated mentorship, curricular flexibility, and outcome-focused research.

#### 4.1.1 Exposure

EM residencies could offer 2–4 week electives in Pain Medicine, ideally in collaboration with affiliated fellowship programs. These rotations allow trainees to explore the field, assess fit, and develop relevant skills not otherwise provided during EM training. For programs without in-house fellowships, offering “away” rotations would be an alternative. EM residency didactic curricula could also integrate longitudinal pain management content aligned with existing blocks (e.g., MSK, neuro, trauma) and feature lectures from faculty in Pain Medicine.

#### 4.1.2 Mentorship

Dedicated mentorship from EM-trained Pain faculty is valuable in supporting applicants through the transition into fellowship. Mentors can share their experiences, provide career guidance, and collaborate with national societies to expand workshops, application advising, and research opportunities. Programs such as the PainConnect EM Workshop exemplify the benefits of cross-specialty mentorship and collaboration [33].

#### 4.1.3 Curricular flexibility

Since fellowship training is condensed into a single year, a self-directed approach is particularly valuable for EM fellows. This includes reflection on prior training, identification of competency gaps, and proactive communication with program leadership. Program Directors can tailor experiences to address areas less emphasized in EM, while reducing redundancy in domains where EM physicians are already proficient (summarized in Fig. 1).

#### 4.1.4 Research

Future research should evaluate competency attainment among Pain Medicine fellows and graduates across base backgrounds. Quantitative competency assessments of incoming fellows, program graduates, and alumni could help define benchmarks and identify areas where reinforcement is needed. Such data would inform curriculum design and promote consistent achievement of competency across varied base specialties. In addition, EM trainees offer a unique perspective on systems-based care, particularly in addressing fragmented transitions, loss to follow-up, and avoidable ED visits and hospitalizations. Future research could examine how EM-trained Pain physicians engage in areas such as transitional pain services, complex high-utilization patient plans, and care pathway innovation, compare these patterns with graduates from other base specialties, and evaluate the broader impact of EM-trained physicians on the multidisciplinary practice of Pain.

## 5 Conclusion

EM is one of the fastest-growing entry points into Pain Medicine, yet it remains an understudied pathway. This paper provides the first systematic, competency-based comparison of ACGME Pain Medicine requirements with five core EM training documents. Findings highlight where EM trainees are well-prepared and where targeted fellowship training is needed, providing a foundation for curriculum planning.

Our analysis demonstrates that EM training overlaps substantially with Pain Medicine in procedural and resuscitative care, including ultrasound-guided interventions, procedural sedation, intravenous access, advanced airway management, and both basic and advanced life support. Additional overlap exists in clinical evaluation, including musculoskeletal, neurologic, and psychological assessment, substance use disorder, and acute pain management. In contrast, gaps remain in the provision of specialized longitudinal care, including advanced pharmacology, imaging, electrodiagnostics, rehabilitation, fluoroscopy-based interventions, and neuromodulation. These findings suggest fellowship curricula should reduce redundancy in acute care and emphasize underrepresented domains.

Beyond the domains captured in our crosswalk, EM training also develops skills in efficiency, prioritization, and systems-based care. Routine exposure to fragmented transitions, high-risk populations, and preventable complications offers EM physicians unique perspectives that may be relevant to inpatient, outpatient, and transitional pain services, including efforts to reduce preventable ED visits and hospitalizations. These contributions warrant further study.

To support integration of EM-trained fellows, structured electives, targeted mentorship, and early exposure to chronic pain management may be valuable. Comparative research on competency outcomes across different training backgrounds could inform curriculum design and help maintain fellowship quality. As the number of EM applicants to Pain Medicine increases, clarifying strengths and limitations will be crucial for sustaining high standards, strengthening the workforce, and enhancing patient care.

## Ethics statement

This article does not contain any studies with human participants or animals performed by any of the authors.

## Disclosure

The author(s) report no conflicts of interest in this work.

## Declaration of competing interest

The authors declare that they have no known competing financial interests or personal relationships that could have appeared to influence the work reported in this paper.
